# Using supraglottic airways by paramedics for airway management in analogue microgravity increases speed and success of ventilation

**DOI:** 10.1038/s41598-021-88008-x

**Published:** 2021-04-29

**Authors:** Jochen Hinkelbein, Anton Ahlbäck, Christine Antwerber, Lisa Dauth, James DuCanto, Elisabeth Fleischhammer, Carlos Glatz, Steffen Kerkhoff, Alexander Mathes, Thais Russomano, Jan Schmitz, Clement Starck, Seamus Thierry, Tobias Warnecke

**Affiliations:** 1 Department of Anaesthesiology and Intensive Care Medicine, Medical Faculty, University Hospital of Cologne, Kerpener Str. 62, 50937 Cologne, Germany; 2 Space Medicine Group, European Society of Aerospace Medicine (ESAM), Cologne, Germany; 3 German Society of Aerospace Medicine (DGLRM), Munich, Germany; 4 Department of Anaesthesiology and Intensive Care, Örebro University Hospital , Örebro, Sweden; 5 Department for Anaesthesiology and Intensive Care Medicine, St. Elisabeth Hospital, Cologne, Germany; 6 Department of Anaesthesiology, Medical College of Wisconsin, Aurora St. Luke’s Medical Center, Milwaukee, USA; 7 Department of Medicine, Goethe University Frankfurt am Main, Frankfurt, Germany; 8 Centre for Human and Applied Physiological Sciences, School of Basic and Medical Biosciences, Faculty of Life Sciences and Medicine, Kings College London, London, UK; 9 Anaesthesiology and Intensive Care Department, University Hospital of Brest, Brest, France; 10 Anaesthesiology Department, South Brittany General Hospital, Lorient, France; 11 Department of Anaesthesiology, Critical Care, Emergency Medicine and Pain Therapy, Hospital of Oldenburg, Medical Campus University of Oldenburg, Oldenburg, Germany

**Keywords:** Environmental sciences, Space physics, Health care, Medical research

## Abstract

In the next few years, the number of long-term space missions will significantly increase. Providing safe concepts for emergencies including airway management will be a highly challenging task. The aim of the present trial is to compare different airway management devices in simulated microgravity using a free-floating underwater scenario. Five different devices for airway management [laryngeal mask (LM), laryngeal tube (LT), I-GEL, direct laryngoscopy (DL), and video laryngoscopy (VL)] were compared by n = 20 paramedics holding a diving certificate in a randomized cross-over setting both under free-floating conditions in a submerged setting (pool, microgravity) and on ground (normogravity). The primary endpoint was the successful placement of the airway device. The secondary endpoints were the number of attempts and the time to ventilation. A total of 20 paramedics (3 female, 17 male) participated in this study. Success rate was highest for LM and LT and was 100% both during simulated microgravity and normogravity followed by the I-GEL (90% during microgravity and 95% during normogravity). However, the success rate was less for both DL (60% vs. 95%) and VL (20% vs. 60%). Fastest ventilation was performed with the LT both in normogravity (13.7 ± 5.3 s; n = 20) and microgravity (19.5 ± 6.1 s; n = 20). For the comparison of normogravity and microgravity, time to ventilation was shorter for all devices on the ground (normogravity) as compared underwater (microgravity). In the present study, airway management with supraglottic airways and laryngoscopy was shown to be feasible. Concerning the success rate and time to ventilation, the optimum were supraglottic airways (LT, LM, I-GEL) as their placement was faster and associated with a higher success rate. For future space missions, the use of supraglottic airways for airway management seems to be more promising as compared to tracheal intubation by DL or VL.

## Introduction

In the near future, interplanetary long-term space missions to Mars and to the Moon will become reality^1^. These missions could last several years (e.g., 3 years to Mars) and will, consequently, be associated with a significant risk of medical emergencies^2,3^. Given the constraints of long-distance and long-duration space missions and the consecutive delay of data transmission, neither evacuation nor telemedical support will be available in a worst-case scenario^1^ urging crews to manage emergencies autonomously under conditions of extreme isolation and with significantly limited equipment. Given these circumstances, the development of clear emergency concepts and protocols including universal and easy treatment instructions is essential^1,4,5^.

Recently it has been estimated for spaceflight that 2.6% of medical conditions potentially require general anaesthesia (GA)^4,6^. Other calculations suggest that one major medical event could occur during a 900 day mission (i.e., a spaceflight to Mars)^7^. Since one single severe event could easily lead to death of a crew member and endanger the mental and physical condition of the whole crew^8^, appropriate emergency protocols are of utmost importance. Airway management is, therefore, a key component during emergency medical treatment^9^ and is not an unlikely event^10^. Although each crew has a specially trained Crew Medical Officer (CMO), the underlying practical training is minimal and the practical experience is comparable to paramedics but not to emergency doctors. Therefore, the risk of mishaps and fatalities during airway management in this setting is extremely high.

A few recent trials comparing several airway devices in microgravity were mainly set during parabolic flight^11–13^. This setting has the significant limitation that the time for each trial is limited to approx. 21 s and, therefore, might be too short to complete airway management—especially in untrained providers.

The aim of the present study was to analyse feasibility, speed, and success of ventilation in different airway devices for airway management by paramedics. To eliminate learning effects, a double randomized cross-over trial was used for securing the airway under free-floating conditions in a submerged setting. The submerged setting and paramedics were chosen since it best parallels spaceflight conditions as well as provider skills. Another important advantage is that airway management is possible without any time restriction.

## Materials and methods

A double-randomized cross-over trial comparing feasibility, speed, and success of ventilation for different airway devices was performed in an analogue microgravity setting. Volunteers used several different airway devices. Randomization for the devices was performed using prepared cards with the planned study sequence. Also, the order of normogravity and simulated microgravity conditions were randomized to eliminate training effects. Randomization of normogravity vs. microgravity was performed by flipping a coin.

### Participants and devices

Participants (n = 20) were trained paramedics (“Rettungsassistent” and “Rettungssanitaeter” in Germany) holding a valid diving certificate. The criteria for inclusion were that the volunteer had to be a paramedic with a valid diving certificate (e.g., Scuba schools international (SSI)—Open Water Diver (OWD), CMAS *, PADI Open Water Diver, ISO 24801-2 (Autonomous Diver), NAUI Scuba Diver). Participants without a valid diving certification or with health-related restrictions were excluded from the trial.

**Figure 1:**
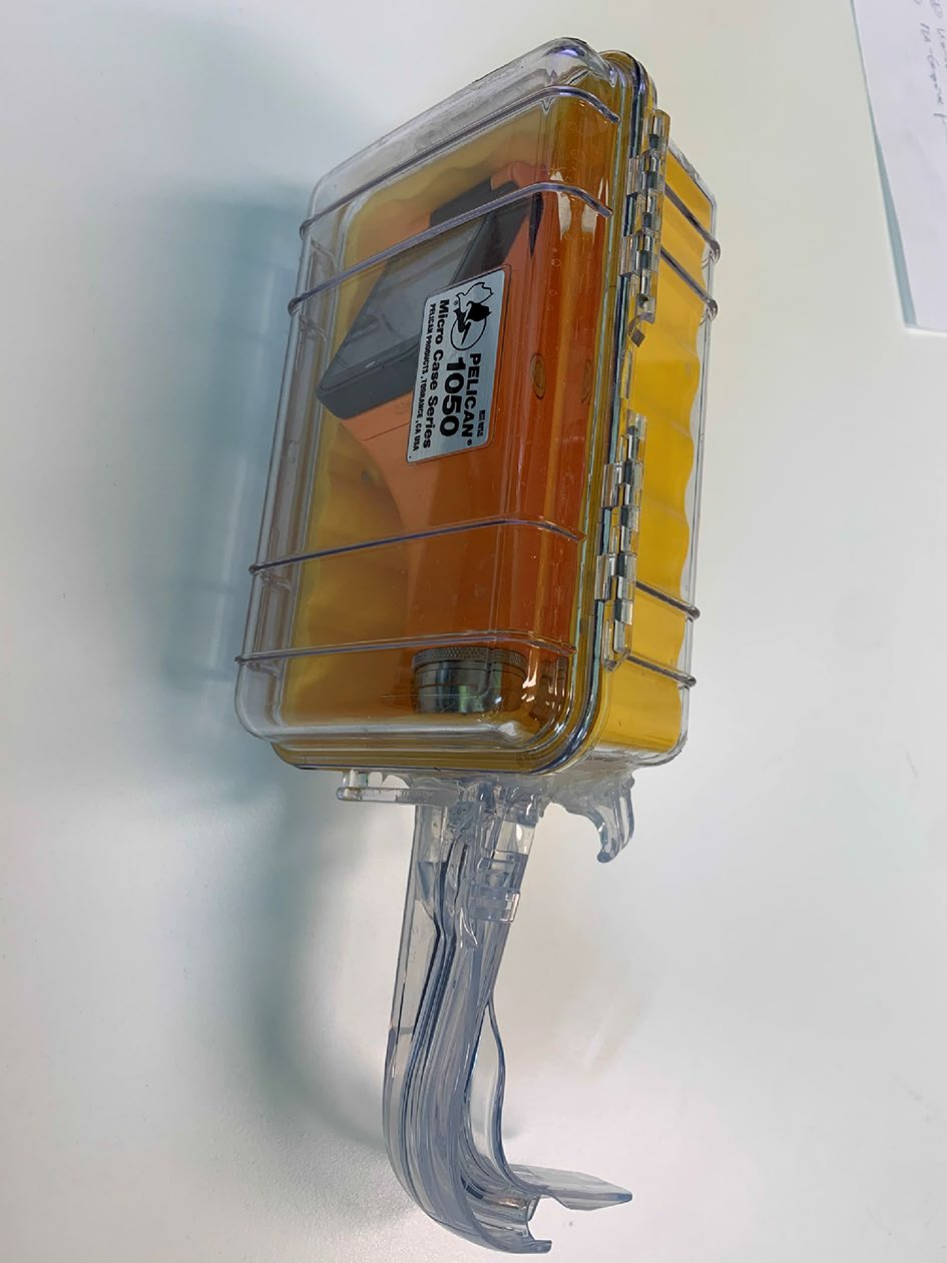
Video laryngoscopy with the PENTAX AWS in a PELICAN 1050 underwater case.

After obtaining written informed consent, participants were asked to fill in a short questionnaire to gather demographic information about the participant’s level of experience for airway management, experience years as paramedic and their total number of dives. Participants had to perform airway management with five different devices in a randomized order:

- Endotracheal intubation by direct laryngoscopy (MacIntosh blade; Heine Optotechnik GmbH, Gilching, Germany) with an orotracheal tube (Magill type; P.J. Dahlhausen Ltd., Cologne, Germany) and introducer,
- PENTAX AWS video laryngoscope for endotracheal intubation (Ricoh Imaging, Tokio, Japan) in an PELICAN 1050 underwater case (Peli products UK, Glossop SK13 6LQ, UK; Fig. 1)
- AURAGAIN, 2nd generation laryngeal mask (Ambu Ltd., Bad Nauheim, Germany),
- LTS-D, 2nd generation laryngeal tube (VBM Medizintechnik Ltd., Sulz am Neckar, Germany),
- I-GEL laryngeal mask (Intersurgical, Sankt Augustin, Germany).

### Setting

**Figure 2:**
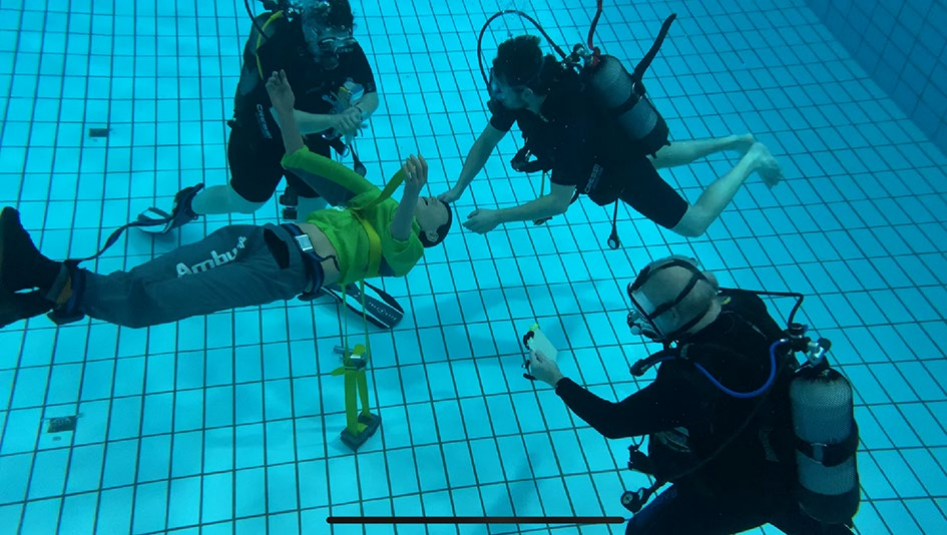
Underwater setting of the trial. The manikin is free floating approx. 1.5 m above the ground level and attached with straps to prevent significant dislocation. Clockwise description: Diver assisting with the devices (left); volunteer paramedic (middle); additional diver/diving instructor for time count and documentation (right).

Airway management was performed using a full-body mannequin (AMBUMAN Airway Wireless, Ambu Ltd., Bad Nauheim, Germany) that was submerged and counterbalanced in a free-floating position approx. 1.5 m above the ground (Fig. 2). For all volunteers, one additional diver, one diving emergency doctor and one dive instructor accompanied the trial, to monitor the setting in case of emergency , for measuring time and for evaluation of success of ventilation. The volunteers had time to achieve neutral buoyancy prior to the trial.

### Measurements

The primary endpoint of the study was successful placement of the airway device. Time count performed by the accompanying diver started once the volunteer took the device in his/her hand and ended with the connection of the ventilation-bag after placement of the device, respectively. Time to ventilation is an often used parameter for comparisons of airway management studies^14^. Since a conventional control of ventilation with air was not possible due to the submerged setting (e.g. pulmonary watering of the mannequin as well as impossibility of using electricity-based equipment), the correct position of the device (ETI by DL and VL) was checked visually by an experienced anaesthesiologist/emergency physician. One additional (paramedic) diver validated the result. Thereafter, a trial to “ventilate” the lungs with water was performed to identify chest movements (all devices). The secondary endpoints were the number of attempts and the duration of placement (time from gripping the device until ventilation).

To analyse the feasibility, speed, and success of ventilation, each trial was limited to one minute. This cut-off value has been set intentionally since it was considered ideal to prevent significant desaturation in real scenarios. All attempts more than 60 s were defined as “insufficient” and were terminated.

### Data analysis

For statistical analyses, data was processed with EXCEL for Mac 16.32 (Microsoft^©^, Redmond, USA). Statistical power calculation was based on previous studies (Keller et al.^15^: 19 ± 3 vs. 33 ± 21 s) as well as own pre-trial tests (underwater: 40 ± 12 s vs. 28 ± 12 s) for estimated differences in both gravity conditions. With Cohen’s d > 0.8, Alpha 0.05 and Power 0.8 the required number of participants was N = 24 (data from Keller et al.^15^) and N = 18 (pre-tests). Therefore, a group size of N = 20 participants was chosen.

Data was checked for normal distribution with Kolmogoroff-Smirnov test and the differences were tested by the unpaired t-test. Results were considered significant if p < 0.05. All findings are presented as means ± standard deviation [p-value] if not stated otherwise.

### Ethics and registration

This study was registered on ClinicalTrials.gov (NCT03848559, date 20/02/2019) and authorized by the ethical committee of the University Hospital of Cologne (19-1069_1, date 01/04/2019). All methods were carried out in accordance with relevant guidelines and regulations.

## Results

**Figure 3:**
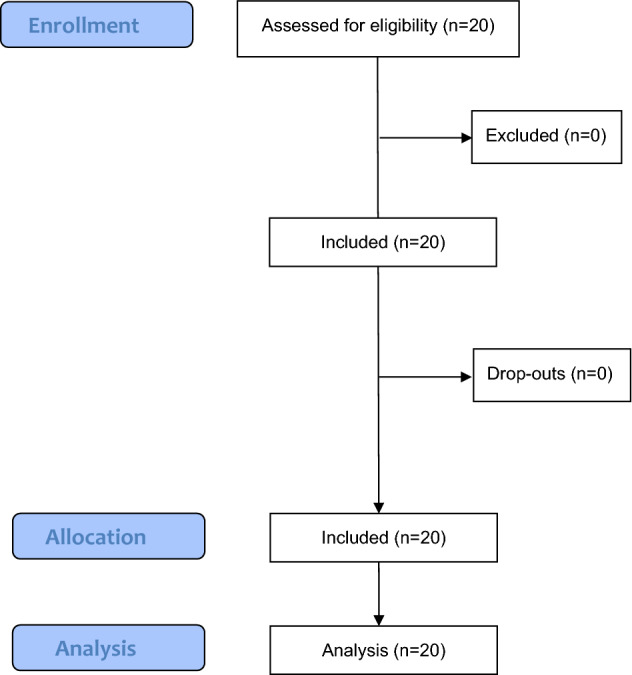
CONSORT 2010 Flow Diagram^37^ of participants.

For the present study, a total of n = 20 paramedics (Fig. 3) participated and performed n = 100 airway management attempts in simulated microgravity (underwater, pool) and n = 100 attempts on the ground (normogravity, control). Paramedics performed airway management either first on the ground (outside the pool) followed by simulated microgravity (underwater), or vice versa, in a randomized fashion. Also, the order of airway devices was randomized. Therefore, each participant performed ten procedures (five in microgravity, five in normogravity).

Three women and 17 men participated in the study. Demographic parameters for female and male paramedics did differ significantly for weight (female: 63.3 ± 6.5 kg vs. male: 84.5 ± 14.1 kg; p = 0.00041) and age (female: 22 ± 2 years vs. male: 32 ± 9 years; p = 0.00052) but not for height (female, 170.7 ± 6.7 cm vs. male, 180.1 ± 8.9 cm; p = 0.05988). All volunteers held a current diving license (OWD: 25%, CMAS*: 15%, CMAS**: 10%, other: 50%) with n = 12 divers with less than 100 dives and n = 8 divers with more than 100 dives.

All paramedics had experience with different airway devices due to their regular employment (means of previous experience, ETI: 20.7 procedures performed; laryngeal mask: 20.1; video laryngoscopy: 1.6).

### Success rate

For all devices tested, success rate was equal or higher during normogravity as compared to simulated microgravity (Table 1). Success rate was highest for LM and LT and was 100% both during simulated microgravity and normogravity followed by the I-GEL (90% during microgravity and 95% during normogravity). However, the success rate was less for both DL (60% vs. 95%) and VL (20% vs. 60%).

**Table 1:** Success rates (within 1 min) [%] in different groups.

Setting	LM	LT	I-GEL	DL	VL
Simulated microgravity/underwater	100	100	90	60	20
Normogravity/ground control	100	100	95	95	60

### Number of attempts

The number of attempts before positive ventilation within 1 min during simulated microgravity was significantly lower for all SGA (each, 1 ± 0 attempts) as compared to tracheal intubation (DL, 1.65 ± 0.93 attempts [p = 0.00345]; VL, 2.25 ± 1.00 attempts [p < 0.001]). Also, simulated microgravity increased the number of attempts for positive ventilation as compared to the use on ground both for DL (ground vs. microgravity, 1.25 ± 0.55 vs. 1.65 ± 0.93 [p = 0.05392]) and VL (1.75 ± 0.85 vs. 2.25 ± 1.00 [p = 0.05017]) but without statistical significance.

### Time to ventilation

Fastest ventilation was performed with the LT both in normogravity (13.7 ± 5.3 s; n = 20) and microgravity (19.5 ± 6.1 s; n = 20). During microgravity, time to ventilation with the LM (23.1 ± 11.5 s) and I-GEL (26.3 ± 11.1 s) were longer but less than tracheal intubation by direct laryngoscopy (41.2 ± 12.8 s) and video laryngoscopy (47.5 ± 8.7 s; Table 2).

**Table 2:** Time to ventilation in different groups. Statistically significant values (t-test) are printed in bold type.

Parameter	LM	LT	I-GEL	DL	VL
Time to ventilation, simulated microgravity/underwater [s]	23.1 ± 11.5	19.5 ± 6.1	26.3 ± 11.1	41.2 ± 12.8	47.5 ± 8.7
	(n = 20)	(n = 20)	(n = 18)	(n = 12)	(n = 4)
Time to ventilation, normogravity/ground control [s]	20.8 ± 8.9	13.7 ± 5.3	14.7 ± 9.0	28.8 ± 10.0	33.5 ± 12.1
	(n = 20)	(n = 20)	(n = 19)	(n = 19)	(n = 12)
p-value (T-test)	p = 0.2426	p = 0.0013	p = 0.0008	p = 0.006	p = 0.0176

For the comparison of normogravity and microgravity, time to ventilation was shorter for all devices on the ground (normogravity) as compared microgravity (underwater). However, for the comparison of normogravity vs. microgravity, statistical significance was not reached for LM (20.8 ± 8.9 s vs. 23.1 ± 11.5 s; n.s.) but for all other devices (Table 2).

## Discussion

In the present study, n = 20 paramedics performed airway management with a total of n = 200 attempts both during normogravity and simulated microgravity. It is, to our knowledge, the largest study so far. The present study is the first to analyse the performance of airway management by paramedics in simulated microgravity and to compare the results to a normogravity control group.

Time to ventilation, and the number of necessary attempts were significantly lower for supraglottic airway devices (LT; LM, I-GEL) as compared to tracheal intubation (DL and VL). Also, success rate was always higher if a SGA was used for ventilation as well as for airway management on the ground as compared to simulated microgravity (Table 1).

Furthermore, as compared to normogravity, time to ventilation was always longer during simulated microgravity and reached statistical significance for all devices except the LM. Also, the number of attempts needed for tracheal intubation was lower when used at the ground (normogravity).

During daily routine in the operation room (OR) and in the prehospital environment, both airway management and endotracheal intubation (ETI) are considered complex and can result in severe complications^16,17^ even when performed by experienced emergency physicians^18^. Time for securing the airway and success rates vary significantly depending on the training, clinical routine and the environment^19–23^.

So far, only four studies on airway management in (simulated) microgravity have been published^15,24–27^. These studies were published in the last two decades. However, in the only studyanalyzing airway management during diving, airway management was performed by anaesthesiologists^15^, and the other two studies used parabolic flight conditions (paramedics and anaesthesiologists)^24,25^.

### Setting

For this analogue model of microgravity, qualified paramedics were used as participants. While parabolic flights offer the possibility to study airway management under real microgravity conditions, they are also limited to 21 s. Therefore, for this study, an underwater analogue model was chosen intentionally since it has no time limitation.

All participating paramedics had previous experience with the airway management devices used in the present study. However, advanced airway management is mostly performed by emergency physicians in Germany and paramedics rely on less anaesthesia training during their basic education. Paramedics as participants were chosen by the authors because they best parallel astronauts with respect to their putative experience in airway management. Whereas anaesthesiologists or emergency physicians have substantially more experience in airway management, the experience of paramedics is likely to be comparable to astronauts. Prior to their mission, astronauts receive basic medical training including airway management (ETI and SGAs). Furthermore, the most likely setting in which airway management will be performed during long-term space missions is during an emergency^4–6,28–30^.

In the present study, a scenario was chosen in which the mannequin was un-fixated, because in the most likely circumstances during space flightairway management may not or cannot be delayed by fixation measures. The delay for fixation on the ISS, for example, is approx. 5–7 min since there is only one position with a stretcher on ISS available. Our aim was to analyse airway management in the very early setting after an emergency and, therefore, a non-fixated scenario was used. Despite earlier airway management, this scenario often results in a lower success rate and longer times for airway management since both the mannequin and the volunteer are free-floating.

However, to prevent the mannequin from diving up or down, and to achieve optimal buoyancy , a strap was used. From our point of view, this does not interfere with the “free-floating” scenario of the present study.

### Success rate

Airway management has a significant complication rate even when performed by trained anaesthesiologists^31,32^ and both within^33,34^ and outside hospitals^35^. Whereas the success rate in the present study was > 90% in all supraglottic devices tested, the success rate during direct laryngoscopy and video laryngoscopy was lower.

However, as compared to the tests in normogravity, the underwater setting lead to a lower success rate, especially for tracheal intubation (DL and VL). Rabitsch et al. found a higher success rate for supraglottic airway devices by paramedics during normogravity and microgravity (combitube, 100% vs. 91%) and a success rate for DL of 86%^25^ but experiments were performed during parabolic flight. The success rate by Groemer et al. for direct laryngoscopy during parabolic flight was lower (33–41%) as compared to the present study^24^. Another study by Keller et al. analysed four different airway devices under simulated microgravity underwater^15^. Success rate in the free-floating condition with DL was 15% only although performed by anaesthesiologists, whereas the rate for the LM was comparable to the present study (97.5%) ^15^. Starck et al. analysed tracheal intubation in novices by direct and video laryngoscopy and found the success rate to be significantly higher with video laryngoscopy (80% vs. 40%; p = 0.006)^27^. In this study, a ground control group in normogravity was also assessed for novices and experts (28% and 80%) as well as during parabolic flight (80% and 95%).

### Time to ventilation

Time to ventilation was defined as the time from grabbing the device to positive ventilation and was limited to 1 min only, as previous data revealed successful placement of both SGA and ET took examiners mostly between 20 and 45 s^15^. In case of a duration time of more than one minute, presence of a difficult airway is high, so that further concepts of difficult airway management have to be considered. In the present study, fastest ventilation was performed with the laryngeal tube both in normogravity and microgravity followed by the LM, I-GEL, DL, and VL. For the comparison of normogravity and microgravity, time to ventilation was shorter for all devices on the ground (normogravity) as compared underwater (microgravity).

All other studies reported so far, also analysed speed of airway management for the endotracheal tube and DL, and Starck et al.^27^ also for VL (Table 3). Concerning the time to ventilation for direct laryngoscopy, time needed was longest in the present study (mean 41.2 s) as compared to all other studies. However, most other studies were performed with experienced anaesthesiologists and during parabolic flight where the maximum time is limited to 21 s. Since the cut-off value was 60 s and tracheal intubation performed by paramedics in the present study, a longer mean time could be explained.

**Table 3:** Comparisons for time to ventilation [s] of other studies as compared to the present study.

Parameter	Study	LM	LT	I-GEL	DL	VL
Simulated microgravity	Present study	23.1 ± 11.5	19.5 ± 6.1	26.3 ± 11.1	41.2 ± 12.8	47.5 ± 8.7
	Other studies	^15^: 33 ± 8 (*)			^15^: 33 ± 21 (*) ^25^: 20 (17–27) ^24^: 18 ± 3 ^27^: 17.4 ± 3.7 and 12.5 ± 5.4	^27^: 15.2 ± 5.6 and 10.9 ± 5.1
Normogravity	Present study	20.8 ± 8.9	13.7 ± 5.3	14.7 ± 9.0	28.8 ± 10.0	33.5 ± 12.1
	Other studies	^15^: 19 ± 2 (*)			^25^: 18 (16–22) ^15^: 19 ± 3 (*) ^27^: 14.8 ± 3.9 and 11.0 ± 4.0	^27^: 15.6 ± 4.3 and 9.9 ± 3.5

(*) This is the only study available with simulated microgravity underwater^15^. Other studies are technically limited to 21 s maximum time due to the parabolic flight.

Concerning the use of a laryngeal mask for airway management in the present study, both the times for normogravity and microgravity match very well to the times reported by Keller et al. in 2000 (Table 3)^15^. Also for direct laryngoscopy, times match better as compared to the other studies performed during parabolic flight^24,25,27^.

Video laryngoscopy is associated with a 92% rescue intubation success rate and is more commonly used than other rescue techniques on the ground^36^. However, video laryngoscopy was found to require the longest time to ventilation with a high failure rate in the present study. In contrast Stark et al.^27^ found the time up to three times shorter as compared to the present study. The underlying reason is not fully clear but it is probable that paramedics are less skilled in using a hyper-angulated video laryngoscope as well as using a video laryngoscope during diving (with the sophisticated water-sealed case; Fig. 1). Both factors may impair handling and, therefore, prolong the time needed. So far, only Starck et al. analysed the use of a video laryngoscope during parabolic flight^27^ and no other study analysed the use during diving.

## Limitations and weaknesses of the present study

The present study has several limitations and weaknesses which should be addressed for proper interpretation of the data. First, the study was performed during diving in a submerged setting. Although this is an established method to simulate microgravity on earth, it has its own limitations which need to be discussed. Water as a medium could affect the way airway management was performed. Especially visual conditions in terms of changed colour, dimmed light or impaired stereoscopic vision may have influenced the ability and speed for airway management. Also, differences in sensory-motor function between diving and spaceflight might be possible.

Second, all participants were paramedics and held a current divers license with ongoing diving experience. However, there are differences in the setting as compared to microgravity during spaceflight. Although we believe thatGerman paramedics resemble astronauts for their manual airway management skills best, there might still be differences in qualification, as an astronaut crew medical officer has 23–40 h of experience and paramedics are usually have more training and more routine in in emergency medicine.

Third and most important, this study was performed in a manikin and not in real humans. It is, therefore, possible that airway characteristics (passing of the tube without lubricant underwater) could be different as compared to living humans. There might be optical or mechanical differences as compared to air which may interfere with performing airway management in terms of success or time.

Fourth, control of correct positioning of the device is difficult in this setting. Whereas ETI by DL and VL was checked visually by the independent anaesthesiologist/emergency physician, this was impossible with SGA devices. Therefore, after each placement manual “ventilation” with the water-filled bag was performed to identify chest movements. One additional (paramedic) diver validated the result. However, there might be an inaccuracy in identification in the underwater setting.

Performing such an investigationduring parabolic flight may perhaps reduce certain influencing factors (e.g., optical view, motion underwater etc.), but—on the other hand—will itself have other significant limitations (e.g., maxiumum time 21 s only for a parabola). In view of the limitation for all analogues, careful interpretation of data in the current study is essential.

## Conclusions

Airway management could have a high priority during emergency medicine procedures in future long-term space missions. In the present study it was shown that airway management with supraglottic airways and laryngoscopy is feasible during simulated microgravity if performed by paramedics. Concerning the success rate and time to ventilation, the optimum technique was using supraglottic airways (LT, LM, I-GEL) as their placement was faster and associated with a higher success rate. In contrast, securing the airway by direct laryngoscopy was more time consuming and was associated with a high failure rate. In case of an emergency, using SGA as first approach seems feasible, in process of resuscitation, safer airway management (e.g. endotracheal intubation with restrained performer and patient) should always be considered.

## Abbreviations

CMAS: Confederation mondiale des activites subaquatiques
CMO: Crew medical officer
DL: Direct laryngoscopy
ETI: Endotracheal intubation
GA: General anaesthesia
ISS: International space station
LM: Laryngeal mask
LT: Laryngeal tube
PADI: Professional association of diving instuctors
OR: Operation room
OWD: Open water diver
RCT: Randomized controlled trial
SGA: Supraglottic airway
SSI: Scuba schools international
VL: Video laryngoscope, video laryngoscopy
